# The multifaceted impact of volunteering in an Israeli neurosurgery department following the October 7th terror attacks

**DOI:** 10.1186/s13584-025-00717-0

**Published:** 2025-10-03

**Authors:** Gabriella Vulakh, Jonathan Roth, Sigal Freedman

**Affiliations:** 1 Department of Emergency Management and Disaster Medicine, School of Public Health, Gray School of Medicine, Tel Aviv University, Tel Aviv, Israel; 2 Department of Neurosurgery, Tel Aviv Medical Center, 6 Weizmann St, 6423906 Tel Aviv, Israel; 3 Gray School of Medicine, Tel Aviv University, Tel Aviv, Israel

**Keywords:** Volunteers, Surge capacity, Mass causality attack, Disaster preparedness, Healthcare system resilience

## Abstract

**Background:**

The paper examines the experiences of volunteers, residents, and leadership in Tel Aviv Medical Center's (TLVMC) Neurosurgery Department following the October 7th terrorist attacks. It explores how volunteers impacted the department's operations during acute medical staff shortages, a surge in patient needs, and a time of profound national and personal crisis.

**Methods:**

Fifteen semi-structured interviews were conducted with volunteers, residents, and management members in TLVMC's Neurosurgery Department to provide a qualitative analysis of their experiences and insights for future disaster preparedness. The findings are analyzed using three theoretical frameworks: Surge Capacity Framework, Resilience Theory, and Volunteer Process Model.

**Results:**

Participants reflected on the overall impact of volunteers in the department — helping to maintain the service’s functionality and operations, filling gaps in staffing, and providing relief and support to staff members — as well as the non-medical contributions such as increasing morale and a sense of solidarity within the community. Volunteers also spoke about the personal impact of this experience, expressing their desire to give back and have a sense of purpose. Participants emphasized the importance of strong leadership, cohesive teams, and the need for pre-disaster planning.

**Conclusions:**

Volunteers provided essential clinical and morale support. They reported meaningful personal experiences and also offered practical recommendations for future disaster volunteering. Embedding community-wide planning and formal volunteer integration can strengthen healthcare system resilience.

## Background

On October 7, 2023, 3000 Hamas terrorists murdered 1163 people, injured 1940 civilians and soldiers, and kidnapped 255 hostages, including 37 children (ages 9 months − 18 years) and 30 elderly (ages 65–86 years) in Israel [1]. In the first two days of the attack, approximately 2000 people were hospitalized country-wide [2]. Since the attack occurred on Saturday, a Jewish holiday and day of rest from the workweek, the hospital systems were at their lowest staffing levels and unprepared for the patient surge. During the week following the initial attack, 3640 people were hospitalized throughout the country, and 170 people were brought to Tel Aviv Medical Center (TLVMC). After two weeks of war, there were 7319 total patients country-wide and 332 patients at TLVMC. Furthermore, many healthcare workers were called for mandatory army reserves. The acute and significant shortage of medical staff and the surge in patient needs necessitated the incorporation of local and international volunteers in Israeli hospitals.

Volunteers are individuals who dedicate their time to perform unpaid work for a specific cause [3]. Motivations to volunteer often arise from a sense of altruism and a deep feeling of duty [4]. During the COVID-19 pandemic, medical students served as a large volunteer workforce worldwide [5–7]. Following September 11, 2001, the United States created The Medical Reserve Corps (MRC) — a national network of community-based volunteer groups — to train and organize volunteers before deployment in emergencies [8]. Retired medical personnel volunteers are also useful when hospitals experience a surge in capacity during disasters [9]. However, while volunteers are critical in mitigating resource gaps, their integration into healthcare systems remains complex. Research examining 33 New York City hospitals’ perception of their local MRC demonstrated that there are still barriers to calling on outside volunteers during emergencies due to the many administrative, legal, and clinical challenges that hinder the use of volunteers [8]. Furthermore, while volunteerism can occur through both formal and informal means, during the acute response phase of disasters, individuals tend to spontaneously emerge for volunteering without a structured or organized process in place, sometimes becoming more of a burden than help [10, 11].

In the aftermath of the October 7th attacks, many medical volunteers took time off from their regular jobs to assist hospital department operations, even if their current work was not in the hospital setting or medical field (despite prior training), or their previous training was in a different specialty. This paper examines the experience of TLVMC’s Neurosurgery Department following the October 7th attacks, exploring how volunteers impacted operations during acute medical staff shortages, a significant surge in patient needs, and a time of profound national crisis. Whether caused by war, pandemic, or natural disaster, this study offers valuable reflections from volunteers, neurosurgical residents, and management on utilizing volunteers and improving future disaster preparedness in Israel and globally.

### Theoretical frameworks

Three theoretical frameworks were used to guide the study in understanding the multifaceted role of volunteers following the October 7th attacks: Surge Capacity Framework, Resilience Theory, and Volunteer Process Model (VPM). The Surge Capacity Framework provides a critical lens for understanding how hospitals expand resources to meet sudden increases in patient demand during disasters [12]. This framework defines surge capacity across four essential domains: Space, Staff, Supplies, and Systems [13]. Unlike other disaster models, Surge Capacity explicitly focuses on resource amplification in healthcare — including human resources. For example, the framework highlights that a key strategy is augmenting personnel by calling in off-duty staff, retired professionals, or volunteers [14]. Surge Capacity Framework can be used to interpret how volunteers filled staffing gaps. In the case of TLVMC, medical volunteers expanded the Staff domain, helping the hospital absorb an influx of patients. Additionally, effective surge response requires organizational systems for volunteer integration, such as credentialing, training, and assigning tasks. Such coordination fits into the Systems domain and shows how institutional processes influence volunteer deployment. This framework is well suited for the study because it focuses on hospital preparedness and response, as well as staffing logistics. It guides the study in interpreting why volunteers were mobilized and how volunteer recruitment and deployment mitigated staffing shortages under crisis conditions.

Resilience Theory adds a complementary perspective by focusing on adaptation and continuity of care. It provides a framework for understanding how individuals and systems adapt to and recover from adverse events or crises [15, 16]. In healthcare, resilience refers to the capacity of hospitals and staff to absorb shocks, maintain core functions under extreme conditions, and emerge stronger [17–19]. Using this lens, the study analyzes how TLVMC’s Neurosurgery Department adapted to the crisis, maintained patient care and operations, and whether they emerged stronger through incorporating volunteer support. The framework supports the interpretation of findings both in terms of system flexibility and recovery, and individual resilience physically and psychologically. The study also considers whether the department learned new practices or became more adaptive and cohesive as a result of the crisis. Ultimately, Resilience Theory guides the study in examining how TLVMC’s Neurosurgery Department managed to “bounce back” and reorganize under stress.

Finally, the Volunteer Process Model (VPM) provides a structured understanding of why individuals choose to volunteer, how they integrate into organizations, and what sustains their involvement [20]. The motivational phase of VPM explains how personal values, social responsibility, and psychological rewards drive individuals to volunteer in crises. In applying VPM, the study also considers the Israeli context of war. VPM guides analysis of the specific motivations behind TLVMC’s volunteers, which may include not only universal values such as altruism and professional duty, but also strong national and ideological commitments. VPM also highlights how the experience of volunteers — their integration within hospital systems and satisfaction with their volunteer activity — can either enhance or hinder their ability to contribute effectively [21]. The final phase of VPM addresses sustained volunteerism and how volunteer efforts can be optimized for better involvement of volunteers, extended duration of activity, and benefits gained to the organization [22]. This framework emphasizes the importance of structured recruitment, training, and retention strategies. VPM provides a nuanced lens to the human component of this study, connecting personal motives to organizational outcomes.

Together, these three frameworks serve as a strong foundation for the study. Surge Capacity Framework describes how volunteer staffing addressed the immediate emergency, Resilience Theory helps explain how incorporating volunteers supported organizational response and recovery, and VPM gives insight into who the volunteers are and what factors led them to contribute. These frameworks are particularly suited for studying volunteerism in a war-time healthcare setting and aid in interpreting the study’s findings.

## Methods

### Participants & study design

The study was conducted following the Consolidated Criteria for Reporting Qualitative Research (COREQ) guidelines [23]. Semi-structured qualitative interviews were conducted throughout January 2025 with TLVMC Neurosurgery Department management members, neurosurgical residents and both local and international volunteers. Qualitative interviewing, based on the phenomenological approach, was chosen to allow participants to share their lived experiences and reflections thoroughly [24].

A total of 15 interviews were conducted in English (three management members, three neurosurgical residents, and nine volunteers). The interviewer was author GV, a master’s student in disaster management with prior experience conducting interviews as a science journalist. She worked as a researcher in TLVMC’s Neurosurgery Department during the study as part of an internship for her master’s program. GV was mentored by a professor with expertise in disaster management and qualitative interviewing.

Participants were sampled iteratively and purposefully to capture diverse perspectives and backgrounds. A sample of nine out of the 13 total volunteers, and three out of the five residents who were present in the department during the study period were asked to participate. All participants responded affirmatively. Selection criteria accounted for variation in professional roles and seniority, with participants ranging from neurosurgical and nursing staff in management positions to neurosurgical residents. For the volunteer participants, researchers ensured diversity in geographic location (local and international) and medical background or work experience (neurosurgeons, physician assistants, medical student, medical doctor working as a data scientist, and medical doctor pursuing a PhD). These criteria were chosen to encompass a broad range of experiences and viewpoints. Recruitment continued until variation across these key dimensions was achieved.

Participants were recruited either by email or WhatsApp messaging. Interviews were conducted in-person at TLVMC, via videoconferencing, or by phone call, depending on the location of the participant. Only the interviewer and participant were present during the interviews. Sample size was based on the principle of thematic saturation, defined as the point at which no new codes or themes emerged from successive interviews [25]. The research team met regularly during data collection to discuss emerging findings and assess whether additional interviews were contributing novel insights. Once consecutive interviews yielded no new codes or meaningful variations in existing themes, it was concluded that thematic saturation had been reached.

Interviewees received information on the topic of the study and reason for doing the research, before providing informed consent to being recorded. None of the interviewees declined to participate or dropped out. All interviews were conducted following the semi-structured interview guide (see Table 3). The interview guide was developed based on existing literature surrounding volunteerism and healthcare worker resilience during disasters [4, 26]. While the interview guide was not pilot-tested or changed during the study, all authors independently reviewed the guide and worked together to refine it prior to the first interview. Interview length ranged from 10 to 40 minutes, depending on the depth of participants’ responses, the number of pivot questions asked, and the interviewee’s role. The interview guide included audience-specific sections, with additional questions designated for volunteers and management members. No repeat interviews were conducted.

### Qualitative analysis

All interviews were audio recorded and transcribed verbatim. GV and SF manually coded transcripts with guidance from the theoretical orientations of thematic analysis and phenomenology [27]. No qualitative data analysis software was used. The phenomenological orientation influenced coding by encouraging a focus on meaning-making, emotional nuance, and the context surrounding participants’ accounts [24]. Thematic analysis further allowed researchers to inductively find patterns across interviews, while remaining attentive to the depth and texture of individual narratives [28]. The coders identified key themes that emerged from the interviews through an iterative process of reading transcripts, coding, and peer debriefing to help refine and clarify the themes. Attention was given to both code frequency and conceptual significance. Themes were identified when at least three interviewees expressed a common sentiment. Field notes taken during interviews, further helped to guide identification of key themes. A coding structure was developed, beginning with broad themes, followed by subthemes, and finally categorization of specific quotes within each subtheme. Although a formal coding tree was not used, this hierarchical approach ensured a systematic and transparent organization of data. The coding process was further guided by the paper’s grounding theoretical frameworks — Surge Capacity Framework, Resilience Theory and VPM — allowing for both preconceived themes and emergent insights. Decisions about theme inclusion were based on consensus among the researchers, ensuring that each theme was strongly grounded in the data and reflected both recurring patterns and meaningful insights.

While the interviewer did not have a previously established relationship with the participants, it is recognized that her background as a master’s student in disaster management could have influenced the research process. To mitigate potential biases, the researchers participated in regular debriefing sessions to reflect on their positionality and its possible impact. The collaborative process facilitated reflexivity by incorporating multiple perspectives into the interpretation of themes and ensuring consistency and transparency in data analysis [29]. Full transcripts were not returned to participants. However, participants had the opportunity to provide clarifications during the interviews, and member checking with select participants was used to validate the accuracy of captured experiences when necessary. Triangulation was further used to crosscheck information across the different participants [30]. By organizing themes from all interviews, the study weaves together a cohesive narrative of participants’ experiences to present the research findings.

## Results

TLVMC’s Neurosurgery Department includes 60-beds of adult and pediatric neurosurgical patients. More than 2,500 surgeries are performed annually. Overall, the department consists of 18 senior physicians, 10 residents, and 1 fellow. At the start of the war, 7 physicians (4 residents and 3 senior physicians) were immediately recruited to mandatory military reserves and were absent for at least 5 months. The fellow returned to his home country shortly after the war began due to safety concerns. One resident also gave birth in the middle of October and was on maternity leave for 4 months. The crisis period lasted approximately 6 months and afterwards became a chronic situation.

During the first 6 months of the war, the department continued to work in a similar surgical volume. Many of the department’s activities are not elective — treating brain tumors, congenital conditions, trauma, and vascular pathologies — all which need to be addressed in a timely fashion and thus, even if postponed shortly after the war began, had to be treated within the next few weeks. During the first few days, the department treated acute injuries amongst civilians and soldiers. Following the first week and into the chronic phase of the war, the profile of injuries continued to include soldiers with various penetrating injuries of the head and body.

13 total volunteers joined the department, including local and international volunteers, neurosurgeons, non-neurosurgeon physicians, physician assistants, and medical students. All interviewees worked onsite at TLVMC during the study period. Volunteers joined the department through two avenues: previous professional connections to the department or TLVMC’s Volunteer Center. Volunteers arrived at the hospital at various time points following the October 7th attacks, ranging from a few days to several months after the event. The duration of their time in the department is outlined in Tables 1 and 2. Volunteer’s medical licenses were approved by the Israeli Ministry of Health prior to beginning work. International volunteers that completed Steps 1 and 2 of the United States Medical Licensing Exam could transfer their U.S. license to temporary Israeli licenses. International physician assistants were approved to do basic procedures under supervision.

**Table 1:** Interviewee demographics: volunteers

Code	Gender	Origin location	Degree	Current position	Volunteer recruitment method	Arrival date	Duration of volunteering
V1	Female	International	Physician Assistant (experience in OBGYN)	PA	TLVMC Volunteer Center	October, 2023	2 weeks
V2	Female	International	Physician Assistant	PA	TLVMC Volunteer Center	October, 2023	8 months
V3	Female	Local	Physician Assistant (experience in pediatric neurosurgery)	PA	Previous professional connection with department	October, 2023	3–4 months
V4	Female	Local	Medical Student	Neurosurgery Intern	Previous professional connection with department	November, 2023	1 year
V5	Male	Local	Medical Doctor (ophthalmology residency)	Data Scientist	TLVMC Volunteer Center	October, 2023	1 month
V6	Male	Local	Medical Doctor	PhD candidate (immunology)	TLVMC Volunteer Center	November, 2023	1 month
V7	Male	International	Medical Doctor	Neurosurgeon	Previous professional connection with department	December, 2023	1 week
V8	Male	International	Medical Doctor	Neurosurgeon	Previous professional connection with department	January, 2024	1 week
V9	Male	International	Medical Doctor	Neurosurgeon	Previous professional connection with department	November, 2023	1 week

**Table 2:** Interviewee demographics: department staff

Code	Gender	Position
M1	Male	Neurosurgeon
M2	Male	Neurosurgeon
M3	Female	Nurse
R1	Male	Resident
R2	Male	Resident
R3	Male	Resident

Volunteers’ responsibilities differed based on their expertise and the needs of the department at the time of their arrival. Volunteers played a crucial role in filling staff shortages and helping the department’s continued operation. Contributions ranged from direct patient care, medical rounding, and surgical assistance to administrative tasks, teaching, and boosting morale. All volunteer activity was completed under the supervision of core staff to ensure that care was equivalent for all patients. Volunteering also had a profound impact not only on the Neurosurgery Department overall but also on each of the participants themselves. Their experiences provide key insights into future disaster preparedness and volunteer utilization during times of crisis.

The following sections are broken down by themes extracted from each interview. Participant’s quotations are marked with a letter (management: “M,” residents: “R,” volunteers: “V”) and a randomly assigned numeral (see Tables 1 and 2).

### Overall impact of volunteers on the department

#### Maintaining functionality

Volunteers helped the department continue its operations smoothly by assisting with essential tasks such as rounds, admissions, and discharges. One PA “brought technical skills … assistance in operations and help with procedures and general rounding (V2)” while another with over 10 years of experience in pediatric neurosurgery had “medical knowledge of the surgery … and was leading the service in terms of patient care … and post-op care (V3).”

However, since PAs are not formally recognized in Israel, “we needed a medical doctor that’s recognized by the state of Israel … to sign the orders and help us (V2).” PAs were “paired with a physician who doesn’t practice medicine but was a licensed provider … he had the credentials and the language, and I had the medical background and together we worked really well (V3).”

While the volunteer doctor who helped with signing orders had shifted career paths outside of medicine, he “was experienced in running a ward while (previously) working as an ophthalmologist (V5).” He worked closely with the PAs allowing them to take the lead “with all of the hands-on work, but … (since) she didn’t have the credentials to do the forms which I could, we teamed up (V5).”

#### Filling gaps in staffing

Volunteers further assisted the department by filling gaps left by staff called to military service or returning home due to safety concerns. “There were just no physicians. That was a major problem … (the lack of staff) was really hard, everybody was aggravated … it was very intense (M3).”

One PA who arrived several days after the war began explained, “when I came into the team, I got the sense of we’re surviving, but we’re dying … we’re in immense need, there’s so much going on, and we lost all of our support because most people were in (army reserves) (V2).”

Another PA added that “their fellow who ran the service went home to another country, and so they were really left without support for the surgeons. The volunteers were able to allow the team to function at a level that they needed without their staff (V3).”

One of the neurosurgeon volunteers summarized the experience as “both about providing operative assistance and helping the broader team with managing. They didn’t have a fellow … so essentially, the role was to step in as what would be similar to a junior attending or neurosurgery fellow. I provided advice and guidance to the few residents who were there who didn’t have a lot of experience in pediatrics, assisted with seeing patients on the floors or consultations in the emergency room (V9).”

#### Providing relief and support

Volunteers helped alleviate the workload of residents and staff by handling day-to-day tasks, allowing the team to function at a higher level (R2). They “helped us a lot … we started to use the volunteers as our second-night call doctors … to give (the residents) some rest, this was a major impact. Also, to handle the day-to-day work, from blood samples to ECG if we were in the trauma bay or surgery … many things you can do without being a neurosurgeon (R1).”

“Whatever the residents did during their day, I would just … shadow and learn as the day went on. No task was too small for me then… I just wanted to help take off some of the burden (V1).”

One resident cited the use of medical students as helpful volunteers. “We had a lot of med students come here and work very hard with us … the challenge is that you have to teach (them) how to do (procedures) in the middle of the night when you are alone … very difficult and very challenging for me, but we have very talented students that learn … and help us (R3).” Another resident added “when you have more people, they can help you, so it reduces the volume (of work). You just have to manage the people since some have more experience than others (R2).”

One medical student said that she “would go to operating rooms, do small procedures like sutures and catheterizations, and do follow-ups (V4).” She added that “the department was excellent and the residents very good” but that “it was a difficult time … they didn’t have time … to do teaching, all the time focused on finishing other things (V4).”

#### Beyond medical contributions

Volunteers’ contributions extended beyond physical or direct medical support (M3). They provided a morale boost and a sense of solidarity. “The fact that these volunteers came and took the trouble to come to Israel on their expenses … says someone cares about you (M1).”

The presence of volunteers created “a new atmosphere (V6)” in the department. “Patients were not exhausted by the daily routine of resident doctors … and were more satisfied with the treatment (V6).”

One volunteer who came three months after the start of the war said, “I’m not so clear that I impacted the department particularly in a surgical or medical way … I just rolled my sleeves up and became part of the team. I rounded with them, and I scrubbed in the operating room, and I was just there. My impact honestly … was more psychological and spiritual … an American senior doctor taking off their own time to come and just physically be there was a tremendous boost in terms of morale … that impact was greater than the actual impact I had in the operating room (V7).”

Another volunteer who came four months after the war began similarly reflected, “I knew I wasn’t needed there to do surgery … Israeli neurosurgery is very developed and high end and world-class… I was lending moral support … I went to different ORs … conferences, I gave a talk at neurosurgery Grand Rounds. (If) whatever encouragement (I) gave them made them feel that (people) on the outside were at least trying to understand what they were going through — if I contributed that in any way, I was happy to do it (V8).”

### Personal impact on volunteers

#### Wanting to ‘give back’

Many participants felt that volunteering was a way to give back to the country. Volunteering “gave me some relief. I felt that … I’m doing something and supporting the country. It gave me more than I even gave the department (V6).”

One volunteer had just moved to the country a month prior and said that this experience “allowed me as someone from outside … to give back. This was my way of contributing to the overall cause for the Israeli people (V3).”

Another volunteer “was torn between my previous experience as a combat fighter … and now as a physician. I kept reminding myself that I’m (supporting) the entire pediatric neurosurgery service, I am doing the right thing (V5).” He added that “we came there knowing that our brothers and sisters needed us … no one gave us nothing, and no one expected anything, just the true urge of assisting in hard times (V5).”

#### Sense of purpose

Volunteering provided a sense of purpose and fulfillment, while reinforcing participants’ values and goals as healthcare providers. One volunteer said, “a lot of my philosophy of being a doctor is very much focused on … the human aspect of our work … I feel like what I did there and what I experienced was very human and it reinforced my feeling about what my purpose is as a doctor, which is not just to do surgery, but to really take care of people and take care of my teammates and lead by example (V7).”

He added that “it’s important for people to realize that our role as physicians is really to take care of patients and to promote health and to heal … medical care is blind to all of these divisions, and I think that we have an obligation to stay above the fray and do what’s right for our patients and for the medical community. That’s the message I would want to impart to other physicians (V7).”

One volunteer said she “was inspired by the people working there. Some people in the middle of the day had to leave to go to a funeral. One of the residents had to go to the (army reserves) on the first day … It was very emotional, but it was also the place that I really felt I had to be. I felt very privileged to be able to volunteer during that time (V1).”

Another volunteer reflected, “it was impressive to see how the hospital was going full tilt … this is just about three months after the war started … and the oft-described resilience of the Israeli people was certainly there and in full display … you got to be tough to make that place work with everything that’s been going on (V8).”

### Advice for future volunteer utilization in disasters

#### Importance of strong leaders, unified team

Volunteers highlighted the department’s strong leadership, flexibility, and direct communication. They reported feeling secure in their roles and part of a unified team. One volunteer, who does not regularly work in the hospital setting, said he initially “felt a bit insecure about treating really hard patients” but that interactions with leadership “came with a safe, good feeling that this is something that I can do (V6).”

Volunteers also described the leadership team as being able to “see the talent and skill sets that we have and apply them so well (V2),” and as having “a vision (V5).”

One of the management members said, “resilience is the flexibility to withstand changes, the flexibility to adapt to wherever reality will take you. It was an extreme situation. It’s very hard to educate for this … you need (people) that can inspire and lead those that are behind them and I think in the neurosurgery (department) that’s the way things were (M1).” Another management member added, “it’s difficult to prepare for such a huge event … but the most important thing is to have a good team behind you (M2).”

Volunteers also emphasized that feeling valued and appreciated by the department “means more to us than we can ever express. Just their plain gratefulness was something that I would have worked there for them, for free forever (V2).” Another volunteer added, “the neurosurgery team was so welcoming to all the volunteers … they really made everyone feel like family and were just so appreciative of anything they could contribute to the team (V3).”

#### Lessons for the future: need for pre-disaster planning

Looking ahead to future disaster preparedness, participants emphasized the importance of planning ahead — from creating a volunteer database and clear expectations for volunteers to logistical support with travel and housing. “No one expects these kinds of scenarios and also having a plan doesn’t guarantee you’re going to use it, but it really is the best practice to plan for extreme cases like these (V5).”

He encouraged hospital departments to consider, “what would you do if you had half of your staff? How are you going to act? What are the department’s most crucial services, and who are the people you need? Have that plan somewhere, and think of it twice a year, because when (a disaster) comes, it’ll be the first place you reach (V5).”

Participants also recommended providing trainings to help familiarize volunteers with the hospital system (M3). “People could do a course, even if it be over Zoom … so that if (they) ever need a backup of medical professionals they have people that they could just call (V1).”

One volunteer added that, “language, even if it’s not fluency, but comfort and proficiency is really important … having a language refresher or rapid online AI … tutor for medical (terms) … would probably increase the availability of interested people to become more helpful (V9).”

Volunteers also noted the importance of verifying credentials (M3) and “streamlining licensing so that in an emergency or crisis situation, people can use their domestic license … and it would be recognized for the duration of the crisis for very specific purposes (V8).”

As a final take-away message, one volunteer applied the need for disaster preparedness to his experience during the COVID-19 pandemic in the U.S. “This only underscored what we saw during COVID-19 … we were screwed. We don’t have 1/1000 of the reserve, the facilities, the national spirit (as Israel) … we don’t have that understanding, national will or capacity … We are very rapidly forgetting that these kinds of emergencies and crises are much more common in our world than we’d like to think. It’s quite a different level of seriousness (in Israel) on an organizational capacity level (V9).”

He added, “make sure that your health care organizations actually have the capacity to provide care in a crisis, whether that crisis be an earthquake or a fire or virus … the organizational lesson is how do you have system resiliency for system crises? From a personal level, we should think about this as physicians, as neurosurgeons (V9).”

## Discussion

TLVMC Neurosurgery Department’s volunteers were instrumental in sustaining clinical operations and bolstering system resilience during crisis. Their contributions filled critical staff shortages when nearly a third of the department’s physicians were mobilized to military service. Volunteers supported the continuation of patient rounds, surgical care, and administrative tasks, while also boosting morale during a period of collective stress. As one management member described, their presence “says someone cares about you (M1).” In line with Resilience Theory, these efforts helped the department absorb, adapt to, and recover from the shock of war. These findings reinforce existing disaster literature suggesting that community-based responses strengthen healthcare system capacities and resilience [31].

Applying the Surge Capacity Framework, this case illustrates how volunteers enhanced both the Staff and Systems domains. Tasks such as administrative duties, assisting with procedures, or signing medical orders — expanded the department’s functional capacity. These contributions relieved pressure on core staff and extended the department’s clinical reach. At the systems level, collaboration with the Israeli Ministry of Health allowed expedited credentialing, enabling volunteers to begin clinical work immediately upon arrival. Within the department, volunteers were integrated into existing workflows, assigned roles based on skill set, and supervised by core staff. These internal adaptations, though largely improvised, demonstrate key principles of surge readiness, including flexibility, strong leadership, and real-time coordination. Such improvisation, resourcefulness, and creativity have also been shown to be indicators of organizational resilience [32].

Volunteering was deeply meaningful for participants. Many described a renewed sense of purpose and community — echoing existing literature [33, 34]. This is consistent with the Volunteers Process Model’s emphasis on personal motivations, emotional outcomes, and sustained engagement. Volunteers described their work as “psychological and spiritual” (V7) and that they “felt very privileged to be able to volunteer (V1).” Another reflected: “(If) whatever encouragement (I) gave them made them feel that (people) on the outside were at least trying to understand what they were going through — if I contributed that in any way, I was happy to do it (V8).” Importantly, even short-term volunteering — one or two weeks (V1, V7, V8, V9) — had a profound impact. These findings underscore the mutual benefit of disaster volunteering: while volunteers support the system, the experience also sustains their own resilience [35].

Subtle differences also emerged between participant groups. Management members emphasized pragmatic and logistical concerns — such as acute staff shortages (“There were just no physicians … it was very intense (M3)”) and the importance of effective teams (M2). In contrast, volunteers highlighted intrinsic motivations, moral imperatives, and a desire for solidarity. Furthermore, both Israeli and international volunteers described a strong sense of duty, but their narratives differed. International volunteers described themselves as offering presence more than technical support: “I knew I wasn’t needed for surgery … I was lending moral support (V8).” Israeli volunteers framed their service as an extension of national responsibility. One physician reflected on his dual identity: “I was torn between my previous experience as a combat fighter … and now as a physician (V5).” This framing evokes the deeply embedded Israeli ethos of collective responsibility, where medical volunteering became emotionally equivalent to military service. These cultural nuances highlight how volunteerism is shaped by local identity and social context.

Finally, this study contributes to the underexplored literature on healthcare system resilience in conflict-affected settings. A recent scoping review identified limited contributions from such environments [36]. TLVMC’s Neurosurgery Department demonstrates how medical volunteers — both domestic and international — were rapidly integrated to support hospital operations under the strain of armed conflict. Uniquely, this study analyzes both the operational contributions of volunteers and the psychosocial meaning they derived from their work. In doing so, it highlights the dual role of volunteerism in reinforcing system capacity and supporting individual resilience. By anchoring the findings in established frameworks of Surge Capacity, Resilience Theory and VPM, the study connects real-world experiences to theoretical insights. The case of TLVMC illustrates how inclusive, well-coordinated volunteerism can strengthen both institutions and the volunteers themselves during times of crisis.

### Health policy recommendations

The findings highlight the need for pre-disaster preparedness that can manifest in improved protocols and organizational strategies. Participants noted that healthcare system preparation could focus on volunteer credentialing, trainings, and maintaining registries of qualified volunteers (M3, V1, V5, V8, V9). Hospitals can establish reserve corps or databases of pre-screened volunteers — similar to an army reserve — supported by structured onboarding, training programs, and legal frameworks for emergency credentialing and liability protection [37–39]. When volunteers come from diverse medical backgrounds, preparation can help ensure a coordinated and unified disaster response.

The findings also indicate that medical students can be helpful volunteers and make up part of the reserve corps, but need the training to be better prepared to assist in emergencies (R2, R3, V4). Medical education policy can promote early exposure to disaster response. Incorporating such training into the medical curricula has been shown to increase students’ preparedness and willingness to serve in future crises [40–42]. Policymakers can encourage partnerships between healthcare institutions and academic centers to formalize these pathways and build a steady supply of skilled, motivated volunteers.

Health policy must also acknowledge the human dimensions of volunteering. Participants reported strong leadership and supportive organizational culture as key in volunteer integration (M1, M2, V2, V3, V5, V6). Health policy should therefore promote leadership training and organizational structures that can adapt in real time. The findings also show how personal and social incentives drive volunteer engagement (V1, V3, V5, V6, V7, V8). Therefore, policies should promote structures that allow volunteers to feel valued and aligned with institutional goals. This includes recognition from leadership, psychosocial support, and communication channels that reinforce a sense of purpose and inclusion [21, 43]. Embedding these principles into disaster preparedness policy will ensure volunteers remain a resilient and reliable component of the healthcare response infrastructure.

Globally, these findings align with the Sendai Framework for Disaster Risk Reduction, which emphasizes strengthening disaster preparedness, and improving response coordination [44]. The framework’s Priority 4: “enhancing disaster preparedness for effective response and to ‘Build Back Better’ in recovery, rehabilitation, and reconstruction” promotes the integration of volunteers into disaster preparedness planning, emphasizing their critical role as stakeholders in disaster resilience [44]. With the support of the World Health Organization, countries can develop international volunteer registries, establish cross-border credentialing agreements, and support international platforms for volunteer mobilization during disasters.

Importantly, these policy recommendations should be tailored to local contexts. The analysis of TLVMC’s experience was limited to one department in an Israeli hospital under wartime. Other settings may have different structures, resources, or leadership dynamics and may require adapted approaches. Ongoing evaluation can guide continuous improvement. Additionally, while each disaster presents unique challenges, advance planning can support system resilience. This study offers transferable lessons for healthcare systems worldwide seeking to improve volunteer integration into disaster preparedness.

## Conclusion

Volunteers consistently reported a meaningful experience, describing how their contributions reinforced both personal and professional purpose. Their motivations — rooted in altruism, solidarity, and a desire to give back — underscore the importance of understanding and fostering intrinsic incentives for sustained engagement. Volunteers delivered critical support to the team, both through hands-on clinical and administrative tasks and by providing a morale boost during a period of conflict.

These findings highlight the necessity of strong leadership, clear coordination, flexibility, and inclusive team culture to integrate volunteers effectively. Healthcare institutions and policymakers can translate these insights into robust emergency preparedness strategies by investing in predisaster training, streamlined credentialing processes, volunteer registries, and psychosocial support mechanisms. In doing so, healthcare systems can be better positioned to leverage the multifaceted impact of volunteers, including enhanced surge capacity and strengthened system resilience, when the next crisis arises.

## Data Availability

The data used and/or analyzed during the current study is available from the corresponding author on reasonable request.

## Appendix

See Table 3.

**Table 3:** Semi-Structured Interview Guide

**Question**	**Pivot questions**	**Audience**
**What is your title and how long have you been working in this field?**	1. What is your current position?	**Everyone**
	2. How long have you been practicing medicine?	
	3. How long have you been in your specialty?	
	4. **Volunteers: **When did you begin volunteering? How long did you volunteer for?	
**How did volunteers impact the department’s function at the start of the war?**	1. How did the integration of volunteers affect the department’s ability to operate during the beginning of the war?	**Volunteers +** **Management**
	2. How were volunteers recruited? What were their specialties?	
	3. What roles were assigned to volunteers, and how did this impact the workload of regular staff?	
	4. Were most volunteers local or international?	
	5. How would you go about creating a reserve of medical volunteers for future disasters? How could they be recruited and trained prior to the disaster to help during a future emergency?	
	6. What lessons from this conflict do you think are important to share with the global medical community?	
**How did volunteers impact the department’s function at the start of the war?**	1. In what specific areas did medical volunteers make the most significant contributions during the war?	**Everyone**
	2. Can you describe the benefits of having these volunteer medical professionals?	
	3. What were the challenges, if any, associated with having these volunteers? What prior preparation would have helped overcome these challenges?	
**What**** impact did the war have on you as a member of the department?**	1. Were you personally involved in the treatment of anyone who was injured due to the war?	**Everyone**
	2. Did knowing that they were injured due to the war or evacuated from war areas affect the way you treat them? If so, how?	
**How**** did the war impact your personal resilience?**	1. Can you describe the emotions you felt during this period?	**Everyone**
	2. How did you take care of your mental health and well-being while working under this emotional toil at the start of the war?	
	3. How has your perspective on your role as a healthcare provider changed since the start of the conflict?	
**Is there anything else you think is important to add?**	1. Is there anything I did not ask about that you would like to share?	**Everyone**
	2. Is there anything else you would like to add?	

## Abbreviations

M: Management
MRC: The Medical Reserve Corps
PA: Physician Assistant
R: Residents
TLVMC: Tel Aviv Medical Center
V: Volunteers
VPM: Volunteer Process Model
